# Neural signatures of reinforcement learning correlate with strategy adoption during spatial navigation

**DOI:** 10.1038/s41598-018-28241-z

**Published:** 2018-07-04

**Authors:** Dian Anggraini, Stefan Glasauer, Klaus Wunderlich

**Affiliations:** 1 Department of Psychology, Ludwig-Maximilians-Universität München, Munich, 80802 Germany; 2 Center for Sensorimotor Research, Department of Neurology, Ludwig-Maximilians-Universitaet München Klinikum Grosshadern, Munich, 81377 Germany; 3 Bernstein Center for Computational Neuroscience Munich, Planegg, Martinsried 82152 Germany; 4 Graduate School of Systemic Neuroscience LMU Munich, Planegg, Martinsried 82152 Germany

## Abstract

Human navigation is generally believed to rely on two types of strategy adoption, route-based and map-based strategies. Both types of navigation require making spatial decisions along the traversed way although formal computational and neural links between navigational strategies and mechanisms of value-based decision making have so far been underexplored in humans. Here we employed functional magnetic resonance imaging (fMRI) while subjects located different objects in a virtual environment. We then modelled their paths using reinforcement learning (RL) algorithms, which successfully explained decision behavior and its neural correlates. Our results show that subjects used a mixture of route and map-based navigation and their paths could be well explained by the model-free and model-based RL algorithms. Furthermore, the value signals of model-free choices during route-based navigation modulated the BOLD signals in the ventro-medial prefrontal cortex (vmPFC), whereas the BOLD signals in parahippocampal and hippocampal regions pertained to model-based value signals during map-based navigation. Our findings suggest that the brain might share computational mechanisms and neural substrates for navigation and value-based decisions such that model-free choice guides route-based navigation and model-based choice directs map-based navigation. These findings open new avenues for computational modelling of wayfinding by directing attention to value-based decision, differing from common direction and distances approaches.

## Introduction

When we navigate in daily life, we commonly do so with a goal in mind, such as going to a restaurant. To reach this destination, we can either follow a route that we know from previous journeys or, alternatively, work out the shortest path using our cognitive representation of the neighborhood. These two possibilities exemplify the route-based and map-based strategies respectively, two prominent strategy adoptions in wayfinding and spatial navigation^1–3^. The route-based strategy, which is a form of response learning, relies on associations between landmarks and turns, as well as memory of travelled distances^1,4,5^. The medial prefrontal cortex, striatum, retrosplenial, and medial temporal regions appear to be important for route-based navigation^5–9^. In contrast, a map-based or place navigation requires knowledge of the spatial relationship between goals, landmarks, or other salient points in space^10–12^. This knowledge can be conceptualized as a cognitive map, which is defined as a mental representation of spatial environment and enables one to acquire, store, code, and recall the relative locations as well as attributes of prior experience in that environment^13–15^. It is acquired by either active searching and exploration, or experiencing the environment using controlled navigational practices, such as exploration using path integration and sequenced neighborhood search^16^. Novel paths may be planned by first searching this map for the best path and then translating this knowledge into a sequence of movements. Map-based navigation engages a distributed system of brain areas including hippocampus^17–20^, parahippocampal^21–23^, and retrosplenial regions^24^. While these studies have identified which neural pathways are involved in each strategy, the computational mechanisms accounting for interaction of route and map-based navigation as well as its neural underpinnings remain a topic of debate, particularly in humans.

In this study, we address these questions by combining a 3D virtual environment, as typically used in imaging studies of spatial navigation, and modelling techniques from the field of value-based decision making. In contrast to previous studies that mostly answered where in the brain different spatial navigation strategies take place, our approach allows us to also investigate how these processes might be solved computationally. In addition, this approach offers an experimental investigation into the utility of modelling techniques from value-based decision-making research in characterizing strategy adoption in spatial navigation.

Similar to the route-based and map-based strategies in spatial navigation, at least two complimentary systems have emerged as dominant behaviors for value-based decision making: model-free and model-based choice systems^25–28^. In the model-free choice, associations are formed between stimuli and actions by reinforcing previously successful actions. Knowledge of the task structure is not necessary for the model-free choice behavior. The model-based choice, on the other hand, relies on a cognitive model of the task structure to evaluate which sets of actions lead to the best outcome. This is done by searching through a map or graph of the task. Extensive neuroimaging studies have suggested computational principles for these two decision systems. These studies also elucidated putative corticostriatal circuits that underlie these computations^29–31^.

Since the computational principles underlying these two value-based choice systems successfully explain a multitude of decision behaviors, they may also provide a useful framework for spatial navigation. While links between spatial navigation and decision making have been previously suggested, mostly in investigations of animal wayfinding^32–34^, they were more general than our hypothesis that the brain shares some of the key computational mechanisms for navigation and for making value-based decisions in a way that model-free choice guides route-based navigation while model-based choice directs map-based navigation. Therefore, we used reinforcement learning (RL) algorithms to model subjects’ traversed routes in the virtual environment. If RL models form the basis of navigational strategies, they should (1) account for subjects’ behaviors when they are free to choose their own navigational strategy, (2) distinguish the degree to which one system is used at each decision point, and (3) explain the role of different brain regions in processing navigational decisions by showing a correlation between BOLD activity and key internal variables of the RL models.

**Figure 1:**
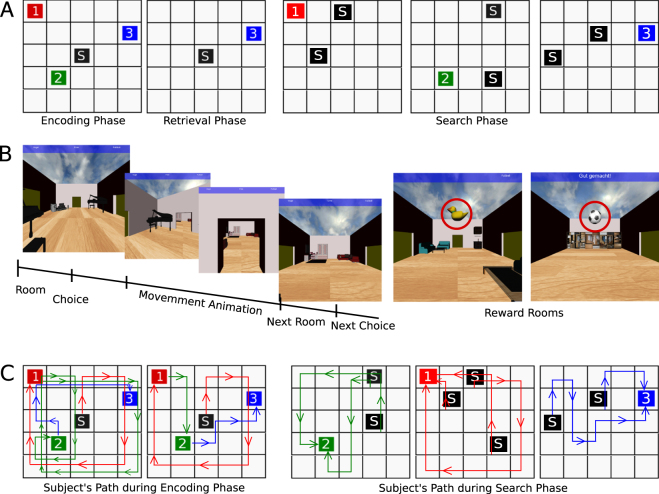
Wayfinding task and behavioral results. (**A**) Layout of the grid-world. The Virtual Reality (VR) environment consisted of a 5 by 5 grid of rooms. Each square represents a room which contained distinct furniture and objects to distinguish individual rooms. Black square represents starting position, colored squares reward locations and the number represents the order in which they need to be found. The wayfinding task consisted of three phases: encoding, retrieval, and search phase. During the search phase, subject had to locate one randomly chosen reward at each trial, each time starting from a different starting position. (**B**) Screenshots of the virtual reality environment. Each room is furnished with distinct objects to allow subjects to distinguish and recognize individual rooms. At each room (decision point) subjects could choose up to three directions (corner rooms had either one or two directions to choose). After a choice was made, an animation was leading to the room in the selected direction; this movement lasted 2.5–3 seconds jittered uniformly. The next room and, if applicable, the reward were presented. (**C**) Path from a representative participant (subject no. 1) who exhibited a tendency towards route-based strategy. During the encoding phase, the subject established by repetition a fixed route from one reward to the other. During the search phase, the subject still followed the established route to reach the reward when it started from a location on that previous route. However, when the subject started from a position which was not part of the original route, she could locate the reward room using the shortest possible path.

To test this hypothesis, we created a wayfinding task in a 3D virtual environment for human subjects. Functional magnetic resonance imaging (fMRI) was then used to investigate how blood oxygen level dependent (BOLD) activity is modulated by computational processes while subjects performed the wayfinding task. The 3D environment consisted of a 5 by 5 grid of rooms, each distinctively furnished to allow subjects to distinguish individual rooms (see Fig. 1A,B). Each subject performed three phases of wayfinding tasks in the same environment. Subjects navigated by freely choosing one of the available doors. Since backtracking (leaving the room from the same door as entering it) was not allowed, rooms in the middle of the grid-world had three doors to choose from, rooms along the outside wall had two doors, and corner rooms had one. First, in the (i) encoding phase, subjects collected three rewards in the same predefined order and from the same starting position over eight trials. This repetition encouraged route-learning during the encoding phase. During the subsequent (ii) retrieval phase subjects collected one randomly chosen reward in each trial from the same initial position as in the encoding phase. Lastly, during the (iii) search phase, subjects collected one randomly chosen reward at a time, from a different starting location. These phases, especially the retrieval and search phases, allowed subjects to use their preferred navigational strategy (further details on the wayfinding task are provided in the SI Method). By analyzing the paths that subjects took, we calculated three navigation indices. These indices describe the degree to which each subject followed previously learned routes (route-based strategy) or engaged in map-based navigation. Based on reinforcement learning models, we also fitted a parameter (ω) that explains whether subjects relied more on model-free or model-based choices during navigation.

In our task, our expectation was that subjects would use a mixture of route and map-based navigation across trials, and that their traversed routes could be well explained by the model-free and model-based RL models. Significant correlations between navigation indices and ω would then confirm that RL models account for individuals’ variability in strategy adoption during navigation. Moreover, we expected that correlates between BOLD activity and RL models’ key internal variables, such as value signals, from the model-free and the model-based choice systems may provide evidence that these algorithms explain how the brain computationally solves the navigation problem.

## Results

### Strategy Adoption during Wayfinding Task

Most participants started early trials in the encoding phase by exploring the environment, often seen by moving from one side of the maze to the opposite until all rewards were collected. Then subjects either found the shortest paths to go between one reward and the next or established a certain route to go from one reward to the other (Fig. 1C). During retrieval and search phases, some subjects (16 out of 27) used the optimal path at least 60% of the time to retrieve rewards. Others chose to follow the route they established during the earlier encoding phase. The latter was a clear sign of employing route-based navigation. Interestingly, during the search phase, when some subjects started on part of an established route they would simply follow it to reach the reward. However, when they started from a position that was not part of their established route, they located the reward using the shortest path. Those subjects might have constructed a cognitive map of the environment but used it only when required to plan a new path. Otherwise, they relented to the cognitively less demanding but potentially longer route strategy. Note that no subject chose exclusively one strategy over the other.

As a first crude measure to quantify strategy adoption in our subjects, we calculated three navigation indices based on: (1) the number of trials in which the shortest path was used (adapted from the work by Marchette and colleague^19^ and referred to as *I*_*PATH*_ in this work), (2) the excess number of steps in comparison to the shortest path required to reach the target (referred as *I*_*STEPS*_), (3) the number of repeated route trials (referred as *I*_*ROUTE*_). Each navigation index was first calculated individually for each phase of the wayfinding task. To get an overview on how subjects performed throughout the entire experiment, every navigation index was then averaged across three phases of the wayfinding task. For both *I*_*PATH*_ and *I*_*STEPS*_, a value of 1 indicates that a subject would have used the shortest path on every trial. In other words, the subject was primarily displaying a map-based strategy. This is because, by design, only subjects who had a good spatial representation of the environment could have reached the rewards using the shortest paths. In contrast, subjects whose scores were close to 0 predominantly used suboptimal long routes, suggesting that they lacked a map-like representation of the environment. The distribution of different values of *I*_*PATH*_, *I*_*STEPS*_, and *I*_*ROUTE*_ revealed that although there are some subjects that showed a strong tendency toward either route or map-based navigation, the majority fell in between (Supplementary Table S1). Further details on the indices are provided in the Methods.

To measure subjects’ general spatial cognitive ability, subjects performed a paper-based Mental Rotation Test (MRT). We found significant correlations between the score on the MRT (*M* = 25.63, *SD* = 9.43, *N* = 25) and *I*_*PATH*_ (*M* = 0.42, *SD* = 0.19, *r(25)* = 0.72, two-tailed t-test *P* < 2.11 × 10^−5^), *I*_*STEPS*_ (*M* = 0.68, *SD* = 0.22, *r(25)* = 0.72, two-tailed t-test *P* < 1.95 × 10^−5^) as well as *I*_*ROUTE*_ (*M* = 0.47, *SD* = 0.18, *r(25)* = −0.72, two-tailed t-test *P* < 2.44 × 10^−5^). These results indicate that greater ability to mentally represent and manipulate objects was associated with higher capability in planning shorter paths. These results are somewhat expected as object-based spatial ability (e.g. mental rotation, spatial visualization) is one of the prerequisites for good performance in map-based wayfinding^35^.

### RL Model Fits to Navigation Data

**Figure 2:**
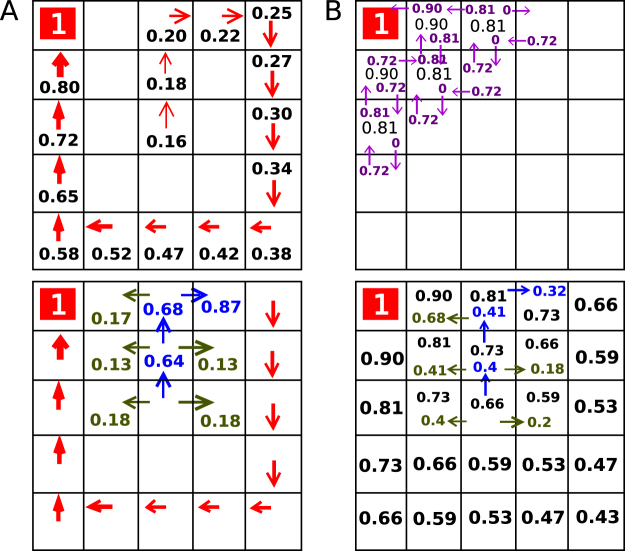
Reinforcement learning models and model fits. The top panel displays action values, showing how valuable it is to move along the route in a certain state; the bottom panel shows the probability of taking certain actions in those state based on the action values. Black numbers are state values, blue numbers are probabilities of chosen action, green values refer to probabilities of other not chosen actions. Note that not all probabilities for non-preferred actions are shown. (**A**) Model free valuation based on the SARSA (λ) algorithm. After reaching a reward this algorithm updates the values only along the traversed path. (**B**) Model-based valuations derived from dynamic programming. The model-based algorithm updates values not only along the taken path, but across the entire grid world.

Similar to navigation in real-life, our wayfinding task is directed toward certain goals and requires decisions about directions along the traversed path. Subjects who used the shortest path could find the reward rooms more quickly and thereby accumulated more rewards. To computationally assess subjects’ navigation strategy at every decision point, we modelled subjects’ choice behavior by fitting three different reinforcement learning (RL) algorithms: model-free, model-based, and a hybrid model formed as a weighted combination of model-free and model-based algorithms. These algorithms exemplify two strategies in value-based decision making: the model-based choice that creates a cognitive representation of the entire environment, and the model-free choice that simply increases action values along the taken paths that previously led to rewards (Fig. 2). The hybrid model assumes that subjects would employ both algorithms at a relative degree, represented by a fitted parameter weight (ω)^25,36^. We performed model fitting for each subject individually and assessed the relative goodness of fit in every phase (for details see Methods, Supplementary Methods, and Supplementary Table S2).

**Figure 3:**
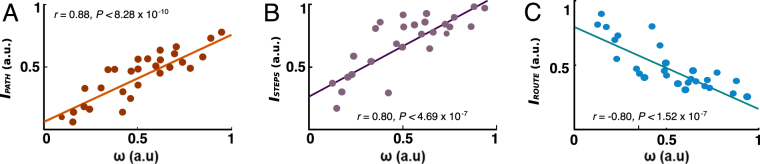
Correlation between navigation indices and ω parameters (n = 27). (**A**) Significant positive correlation between *I*_*PATH*_ and ω parameters. (**B**) Significant positive correlation between *I*_*STEP*_ and ω parameters. (**C**) Significant negative correlation between *I*_*ROUTE*_ and ω parameters for the fMRI experiment. *I*_*PATH*_, *I*_*STEP*_, *I*_*ROUTE*_, and ω are averaged values for individual subject across three different phases of the wayfinding task.

The fitted ω (averaged over three different phases) in the hybrid model (*M* = 0.50, *SD* = 0.23, *N* = 27) significantly correlated with spatial cognitive ability (as assessed by MRT, *r(25)* = 0.54, two-tailed t-test *P* < 0.0035) both *I*_*PATH*_ (*M* = 0.42, *SD* = 0.19, *r(25)* = 0.88, two-tailed t-test *P* < 8.28 × 10^−10^) and *I*_*STEPS*_ (*M* = 0.68, *SD* = 0.22, *r(25)* = 0.80, two-tailed t-test *P* < 4.69 × 10^−7^). These results demonstrate that, across three phases of the wayfinding task, subjects who often took the shortest paths or a relatively small number of steps also showed a tendency towards the model-based choice (Fig. 3A,B). In line with these results, we also found significant negative correlation between ω and *I*_*ROUTE*_ (*M* = 0.48, *SD* = 0.18, *r(25)* = −0.82, two-tailed t-test *P* < 1.52 × 10^−7^). This shows that subjects who repeated learned routes are better explained by model-free RL (Fig. 3C).

While a more optimal map-based or model-based strategy allows shorter paths, the computational effort to plan those paths should be higher compared to route-based navigation. We therefore speculated that subjects who used a higher degree of map-based versus route-based strategies would, on average, show longer reaction times for each choice. Across subjects, we observed a significant positive correlation between average reaction times in subjects’ choices and I_PATH_ (*r* = 0.67, *P* = 1.35 × 10^−4^) as well as I_STEPS_ (*r* = 0.79, *P* = 7.65 × 10^−7^), and a negative correlation between reaction times and I_ROUTE_ (*r* = −0.66, *P* = 1.91 × 10^−4^). We also found a significant positive correlation between reaction times and ω as measure for the relative degree of model-based versus model-free choices (*r* = 0.59, *P* = 0.0012). Furthermore, reaction times tended to be longer the farther away the room was from the reward (linear regression of distance on RT, random effects analysis: *t*_*(26)*_ = 7.242, *P* = 1.08 × 10^−17^*)*, indicating that decisions far away from the reward might be more difficult than decisions close by.

### Neural Signatures of Model-Free and Model-Based Choices

**Figure 4:**
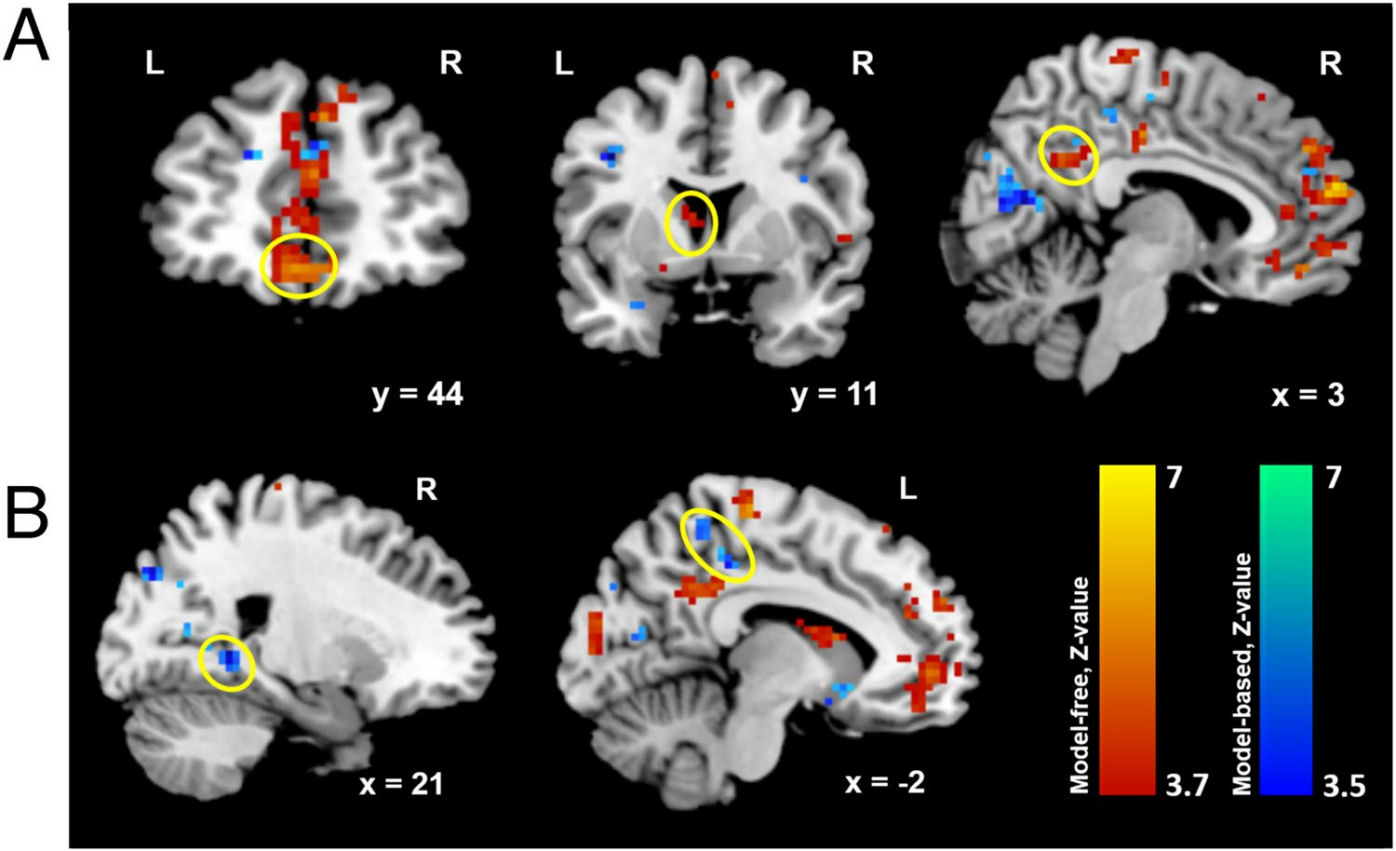
Correlations of model predicted values with BOLD signals. (**A**) Correlates of model-free valuations in medial/vmPFC, striatum, and retrosplenial cortex. (**B**) Correlates of model-based valuation in parahippocampal and medial temporal lobe region as well as the left retrosplenial cortex. Displayed results are significant at *P* < 0.05 whole brain FWE corrected at the cluster level.

We then investigated neural responses pertaining to choice valuations in every room. A key internal variable of the RL algorithms is the value of the chosen action. This value equals the expected reward after a choice has been made^37^ and is a signal that has been reliably detected in BOLD fluctuations over a large number of studies^37–43^. For every decision point, we took the values of the chosen action (i.e. go to the left, right, or straight ahead), calculated separately based on model-free and model-based RL, along the traversed path as parametric modulators for our fMRI data. By analyzing three different phases of the wayfinding task in one GLM, we found that BOLD activity correlated significantly with the model-free value signals along left ventromedial prefrontal cortex [vmPFC, peak: x = −3, y = 47, z = −13] extending to left anterior cingulate cortex [ACC, peak: x = −3, y = 38, z = 5]. Other clusters included retrosplenial cortex [x = 6, y = −52, z = 32] and caudate nucleus [x = −9, y = 11, z = 14]. In contrast, significant correlations with the time series of model-based values were most prominently in the area of right parahippocampal gyrus [peak: x = 21, y = −46, z = 2] extending to hippocampus and bilateral calcarine gyrus. Additionally, activity in right precuneus [x = 27, y = −64, z = 29] and left retrosplenial cortex [x = −12, y = −31, z = 44] correlated with model-based values. See Fig. 4 for the activated areas and Supplementary Table S3 and Supplementary Table S4 for a list of all activated areas. For correction of multiple comparisons, we set our significance threshold at *P* < *0*.*05* whole-brain FWE corrected for multiple comparison at cluster level.

**Figure 5:**
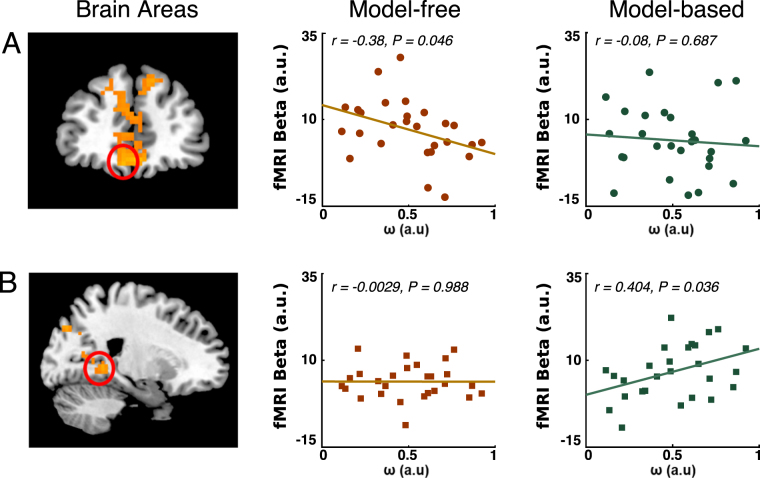
Correlation between subjects’ relative degree of model-based behavior (ω) and β-parameter estimates of the model-free parametric regressor. (**A**) In vmPFC, we found a significant negative correlation between the ω parameter in the behavioral hybrid model and β-estimates in the GLM from the parametric regressor of model-free values. That means, across subjects, the larger the relative degree of model-free choice behavior of a subject, the stronger was her representation of model-free values in the BOLD signal in vmPFC. No such relationship was found for the model-based value regressor. (**B**) In the parahipocampal gyrus, we found a significant positive correlation between ω and β- estimates from the parametric regressor of model-free values. The larger the relative degree of model-based choice behavior in a subject, the bigger was her representation of model-based values in the BOLD signal in the parahipocampal gyrus. No such correlation was found for the model-free value regressor.

If our behavioral models explain subjects’ path choices and subjects’ brain activity represents crucial decision variables in this process, then we would expect that brain activity should be particularly well-explained in those subjects in whom our model also provides a good choice prediction. Specifically, model-free values should explain BOLD activity in vmPFC particularly well in those subjects for whom our hybrid model indicated a large route-based contribution. Similarly, brain activity in the parahippocampal/hippocampal area should be particularly well explained by the model-based values in those subjects in which the hybrid model indicated a large map-based contribution. Across subjects this would be expressed in a relationship between strategy choice (represented by the ω parameter) and strength of the activity pertaining to the parametric value signals of the corresponding model (β-parameter estimates in the general linear model) of the fMRI analysis. Consistent with our conjecture, we found a significant negative correlation (*r(25)* = −0.386, two-tailed t-test *P* = 0.046) between ω from the behavioral model and β estimates in ventromedial PFC for model-free value signals (Fig. 5A). Note that a smaller ω indicates a larger degree of route-based influences. As expected, we did not find a correlation (*r(25)* = −0.081, two-tailed t-test *P* = 0.687) between ω and the β estimates for the model-based value signals in the medial PFC (Fig. 5A). Similarly, we found a significant positive correlation (*r(25)* = 0.404, two-tailed t-test *P* = 0.036) between ω and the extracted parameter estimates of the model-based value signals in right parahippocampal gyrus (Fig. 5B). Again, we did not find a significant correlation (*r(25)* = −0.0029, two-tailed t-test *P* = 0.988) between ω and parameter estimates of the model-free β estimates in the parahippocampal area (Fig. 5B). This confirms that our RL models explain a larger proportion of the fluctuation in the neuronal data in those subjects whose choices are well explained by the respective model. That is, the more subjects lean towards one navigation strategy, the more clearly the computational value signals of the corresponding decision mechanisms are seen in either the vmPFC or the parahippocampal area. Our test was not sensitive enough to show a significant difference between the two correlations in both parahippocampal gyrus (*Fisher-z* = 1.494, *P* = 0.068) and mPFC (*Fisher-z* = −1.129, *P* = 0.129). What we showed was that, across subjects and within a certain brain region, there is a relationship between strategy choice on the behavioral level and the representation of the corresponding value signal in the brain. In effect, these results show that BOLD activity in vmPFC and parahippocampal area reflected model-free or model-based valuations in proportion matching those that determine subjects’ use of route-based or map-based navigation.

We also tested several alternative hypotheses for putative signals that the brain might keep track of during navigation. One such hypothesis is that the brain combines values from the model-free and model-based using the relative weight parameter ω. We tested for this by looking for neural signals pertaining to values of the hybrid RL model, which is a weighted combination of model-free and model-based values. These signals did not survive cluster level correction for multiple comparison.

We further explored the idea that the brain encodes the distance to goals during navigation. We found that while activity in the precuneus positively correlated with the distance to goal, activity in fusiform and caudate increased as subjects approach the goal. Consistent with previous studies, activity in precuneus may be indicative of a spatial updating process during navigation^44,45^. Because there might be a functional relationship between distance to goal and choice values, we also tested if our earlier results for model-based and model-free choice values are indeed correlates of value signals, or could potentially be only a spurious result of this relation. To test for this, we estimated separate GLMs containing both value signals and distance parametric regressors without orthogonalization, letting them directly compete for variance. Any remaining activation in this analysis can be attributed uniquely to one or the other regressor. Importantly, we still found a significantly correlated BOLD activity with model-based and model-free value signals in medial temporal region in this design (see Supplementary Fig. S6).

## Discussion

We show that human subjects are adept at flexibly employing route-based and map-based strategies while navigating in a wayfinding task. Their choices in each room correlated well with the fitted parameter ω from the hybrid model, which is a mixture of model-free and model-based RL algorithms. The computational processes of model-free and model-based RL were also represented at the neural level. Model-based valuation during map-based navigation strongly modulated activity in parahippocampal and medial temporal lobe (MTL) areas, while BOLD responses in striatum and vmPFC pertained to model-free valuations. Furthermore, we found a direct link between the degree to which subjects used one navigation strategy and the neural representation of value signals associated with the corresponding decision mechanisms.

Converging evidence from both animal and human studies has suggested that navigation predominantly relies on two types of strategy adoption generally termed as route-based versus map-based navigation^46,47^. In most previous studies on spatial navigation subjects were constrained to one navigation strategy, such as passively following a certain route during the encoding phase^5,9,12^. Our encoding phase is different in that we allows subjects to explore. This feature is helpful to elucidate the computational mechanisms accounting for route and map-based navigation. Because subjects are not constrained to one strategy over the other, we can observe the use of both strategies in the same environment. While our encoding phase emphasized the formation of a route between a single starting position and a sequence of three reward locations, it simultaneously allowed the formation of a map-like representation over time because subjects could freely move within the environment. The retrieval phase then allowed further memory formation of how every target location can be reached from the starting position.

To our knowledge, one previous neuroimaging study on value-based decision making has attempted to characterize neural and computational substrates of reinforcement learning using a wayfinding paradigm^48^. Albeit the task created for their study adopted a grid-world configuration commonly used in human navigation, their research questions were more concerned with value-based decision making. For instance, their task included visual cues that constantly informed subjects about the location of the target. Moreover, throughout the experiment, there was an ongoing reconfiguration of the grid-world and occasional random teleportations of the subject. These features are useful to study neural mechanisms of reinforcement learning in a probabilistic environment but preclude investigation of wayfinding using optimal or repeated routes. Consequently, in comparison to this study, our task mimics more of an everyday experience. This allows us to study human spatial navigation where subjects can combine and switch between different strategies depending on which is optimal in each situation.

In the present study we make no claims about the neural mechanism of how the brain compares values of the available choices in every room. We therefore only tested for a neural representation of the value of the chosen action because this post-choice value component has been consistently shown in numerous studies to be a reliable signal present in the brain. It is possible that during the choice, the brain also represents value components of the alternative actions in each room either as sum or differences between values^49^, or that the representation might change as a function of time from one to the other during the decision process^50^. Since subjects have to compare multiple alternative actions and consider multiple navigation strategies, testing for a representation of values of the not chosen actions would be more difficult in the present study. Note, however, that the signal component of the chosen value is also part of an aggregated signal such as a prediction error, a sum, or difference of action values^31^ and is therefore a very robust signal in BOLD data.

The properties and neural correlates of route and map-based strategies have been intensively investigated in both humans^46,51^ and animals^52^. Although the terminology has evolved, the route-map dichotomy is still valid. Previous studies have also investigated the emergence of route vs. map knowledge and suggested that landmark knowledge (necessary for route navigation) and survey knowledge can be acquired in parallel^53^ and that preferential engagement of route and map-based navigation may be a dimension along which individuals vary^19,54^. In the neuroimaging literature, some results strongly support the route-map dichotomy^1,3,8,9,20,55^, other data reveals overlapping or common activations, suggesting that in some cases, the dichotomy may not be so clear cut^2,5,11,56^. Our data supports not only a functional separation in wayfinding between medial prefrontal cortex for route based and medial temporal areas for map based strategies but also include common activations such as in the retrosplenial cortex.

In the parahippocampal and MTL area, we observed activations related to choice values that are likely correlates of model-based choice computations. The hippocampus has been shown to play a key role in processing relative spatial and contextual information, as well as in encoding cognitive maps of the spatial environment and the current position in space in both animals^14,57,58^ and humans^59–61^. These processes are crucial for our wayfinding task for forming a map-like representation of the environment. To do this, subjects need to not only keep track of their current location relative to the reward location but also to integrate and transform spatial information into survey knowledge (or an allocentric reference system). Moreover, the parahippocampal gyrus responds selectively to visual scenes depicting places^62^ and is also specifically involved in the retrieval of spatial context compared with non-spatial context^63–65^, identification and retrieval of landmarks^66,67^, as well as spatial relationship and relevance of landmarks encountered during navigation^8,68–70^. A coherent map-like representation of a complex environment, such as the one used in the present study, requires the ability to identify and retrieve different landmarks as well as to form links between landmark identity and the layout of local areas. Consequently, model-based computations in the parahippocampal and MTL area during our wayfinding task could be indicative of searching and planning within a mental representation of the virtual environment to find the shortest path to reach the goal.

For the neural correlates of model-free choices, we observed activations in vmPFC and caudate nucleus, which are consistent with previous studies in animals and humans^26,59,71^. Our model-free algorithm assigns values to subjects’ choices along the taken path. This approach emphasizes the character of route-based navigation including temporal relations between landmarks and sequences of turns^2,8,9,72^. In addition, different studies have proposed that vmPFC encodes stimulus-reward associations^25,73–77^. Thus, by representing model-free valuation, the brain may recall previously cached landmark-action associations. Activity in the vmPFC has also been observed in spatial working memory tasks as well as during retrieval of information about order and context^78–81^. Recent studies have found grid-cell coding in the human brain^61,82^. Note that the findings of grid cells in medial prefrontal cortex do not contradict our results here as we investigate different brain mechanism: while grid cells are most likely used to store a cognitive representation of the environment, we examined possible computations how the brain might find routes within such a representation based on reward values. Due to our parametric analysis, activated areas in the present study highlight only regions where BOLD activity correlates with specific decision value signals and not areas generally involved in spatial navigation or in forming grid-like representations of the environment. In rats, the dorsolateral striatum (DLS) has been shown to be involved in a spatial learning process that relies on rewarded stimulus-response behavior, i.e. model-free choices^34,83^. In other words, neural activity in this region correlates with learning of turns and response to stimuli, which is the hallmark of route-based navigation. In humans, several fMRI studies report that the caudate is activated during route-following and route-recognition tasks^1,5,9,19,84^. Thus, we argue that the encoding of model-free valuations in the vmPFC and left caudate nucleus during our navigation study may reflect computational mechanisms necessary for encoding of relations between landmarks as well as tracing of sequence of turns and places, which are crucial for route-based navigation.

The retrosplenial involvement in navigation has been demonstrated in electrophysiological, neuroimaging, and lesion studies^9,20,21,85^. This area is anatomically closely linked to various medial temporal regions and mid-dorsolateral prefrontal cortex. Patients with retrosplenial lesions have been reported to be unable to form or recall links between landmark identity and couldn’t derive navigational information from landmarks^24^. These two processes are crucial to route-based navigation. Moreover, neuroimaging studies reported performance dependent activation in retrosplenial cortex during mental navigation^85^. Other studies suggested the correlation of retrosplenial activation with the amount of survey knowledge acquired following learning the spatial relation in an environment^9,20^. These findings, along with the modulated activity in this region by both model-based and model-free regressors, support its prominent role in processing landmark information and using landmarks to navigate and discern space. This role is important for both route and map-based navigation.

Since our subjects were not confined to one navigation strategy or the other, they could apply both strategies during the experiment. One might speculate that using such a mixture could result in integrating information from both model-free and model-based computations in a weighted manner for every choice. In our experiment, however, we did not find direct evidence for such an integration as there was no significant correlation between value signals of the hybrid model and BOLD activity. This would support the hypothesis that route and map-based evaluations are not necessarily integrated on every decision point. Instead, we suggest there were trials when subjects relied heavily on either route or map-based strategy to guide their choices during navigation. Note, however, that by design, our hybrid model cannot finally discriminate between both hypotheses. Either way, our calculation of a behavioral weight of model-based versus model-free computations is still meaningful since ω is fitted over all trials and indicates an aggregated relative degree of model-based versus model-free choices over the course of the experiment.

In our experiment, the target rooms that subjects had to find contained a reward (subjects were paid according to how many targets they found) that we could use to update RL values. Therefore, one could argue that we only observed RL learning because of the rewarding nature of the task and this process might not be causally linked to making navigational choices. To rule out the influence of an external monetary reward on our results, we conducted an additional behavioral experiment. The wayfinding task was similar to the one presented here but without monetary rewards. We found no differences in subjects’ choice or navigation behavior (see Supplementary Results, Supplementary Methods, and Supplementary Fig. S5 for details and results). On a neural level, even in situations without explicit reward, goal-directed navigation requires the brain to represent the goal location as some form of intrinsic reward and the brain employs the same reward learning machinery whether rewards are real or fictitious^86,87^. Moreover, if our results were only due to the value of reward, we would expect that the observed brain activations would only mirror the one found in pure decision making. Here we find BOLD activity pertaining to value-based computations in medial temporal regions that are not commonly associated with RL but frequently reported during spatial navigation. All this taken together, it is very likely that our findings indeed form the bases of wayfinding towards the goal state.

In conclusion, our findings show that successful navigation requires the ability to flexibly integrate different strategies depending on the given situation. Importantly, our results also suggest that the neural computations during employment of these strategies might be algorithmically described by RL models. Such a link between value-based decision making and spatial behavior seems plausible given the central role that choice has played in navigation from the beginning of the evolution of central nervous systems.

## Methods

### Participants

27 right-handed females, 20–29 years of age, participated in the wayfinding task while undergoing functional Magnetic Resonance Imaging (fMRI). The decision to recruit only one gender was based on the aim to reduce variance in performance and strategies across subjects and thereby increase power of the tests. This is a common practice in psychological experiments that are designed to study general population mechanism compared to individual differences within the population^88^. While this may limit generalizability of our findings here to men, we are currently conducting a follow up study that includes participants across different gender and age groups that will address these issues. Two additional participants were not included in the analysis because they did not complete all trials of the experiment. All participants had normal or corrected-to-normal vision and no history of either neurological/psychiatric illness or any other contraindications to the MRI environment.

### Experimental Procedure

The study was approved by the ethics committee of the Medical Faculty of the Ludwig-Maximilian-University Munich, all relevant ethical regulations and guidelines were followed. All participants provided written informed consent before the experiment and were paid a compensation of 25 to 30 EUR based on the number of collected rewards during the experiment.

The core of our wayfinding task is a Virtual Environment (VE) consisted of a maze, divided into different rooms in a grid-like manner. Each of them was connected to the adjacent room and furnished with unique set of furniture. Thus, each room is different from all other rooms. While inside one of the room, no information from the neighboring room could be perceived. Three of these rooms are designated as reward rooms. Each reward room contained one of three target objects that subjects had to find while navigating through the maze.

The complete experiment consisted of one practice session and one fMRI session. The practice session was conducted three to five days before the fMRI scanning session. The VE for the practice session consisted of 4 by 4 grid of rooms (16 in totals) with different furnishing than the one we used for the fMRI session. The VE was presented on a 24-inch computer screen.

For the fMRI scanning session, the VE consisted of 5 by 5 grid of rooms (25 in totals). Participants completed one training phase and three test phases (encoding, retrieval, and search phase). During the training phase, right after subjects enter the MRI, they were instructed to freely explore the 5 by 5 grid without any rewards present. Thus, subjects could learn to move with the button press and get comfortable with the stimulus material.

In each trial of the encoding phase, participants were instructed to find three rewards in exact order. This was ensured by making each reward visible only if the previous reward had been found. Once participants had found all three rewards, they were brought back to the same starting position. They then had to perform the exact same task. In total, subjects performed 8 trials, i.e. navigating from the starting position to reward 1, then reward 2, and finally reward 3, during the encoding phase. The task design of a consecutive reward sequence in the encoding phase facilitated route learning.

In each trial of the retrieval phase (navigating from the starting position to a designated single reward), subjects were asked to find one reward at a time for a total of 15 trials. The task design of this phase was well suited to differentiate between route and map strategies. When ask to find reward 2, a subject using a perfect route-based strategy would first visit reward 1 and then go from there to reward 2. On the other hand, a subject using an ideal map-based strategy would go directly from starting point S to reward 2.

During each trial of the search phase, subjects were instructed to find one randomly selected reward at a time, i.e. only the reward that was indicated on the screen. This time, however, the starting position for every new trial was different from the one they encountered during the encoding and retrieval phases.

In addition to the wayfinding task, participants also performed a paper-based Mental Rotation Task (MRT).

For clarity, we summarize a few terms used in this study:

Phase: the game type played by the subject: encoding, retrieval, and search.

Run: the fMRI run (the scanner was stopped between runs for subjects to talk with experimenter and take short break). Note that our encoding phase consists of 8 trials and was conducted within two fMRI runs (4 trials in each run) to give subjects time to rest and minimize head motion. The retrieval and search phases were each conducted in a single fMRI run.

Trial: the sequences of actions from the starting location to the reward location. During the encoding phase, this means from start to reward 3 via reward 1 and reward 2. During retrieval and search phases, a trial means from start to whichever single reward was to be located.

Set of trials: group of all trials where subjects need to find a certain reward, across all task phases (e.g. all trials where subjects had to find reward 2 from encoding, retrieval, and search phases).

### Navigation Indices

To quantify strategy adoption during the wayfinding task, we calculated three navigation indices. For each navigation index, we calculated the index separately for each phase of the wayfinding task (adapted from a study by Marchette and colleague^19^). We first measured (1) the number of trials when subjects used the shortest path to find the rewards, (2) the steps taken to complete one trial in excess of the minimal number of steps, and (3) number of trials where subjects repeated learned routes.

#### I_PATH_ and I_STEPS_

We then calculated the proportion of trials with the shortest path to the total number of trials in a respective phase

1 $${I}_{PATH}=\frac{{n}_{shortest\_trial}}{{n}_{total\_trial}},$$

*where n*_*shortest_trial*_ is the number of trials when subjects used the shortest path to find the rewards and *n*_*total_trial*_ is the number of trials in a given phase.

Alternatively, we determined a navigation index based on calculating the excess steps in each phase. We then defined this index as

2 $${I}_{STEPS}=\frac{{n}_{{\rm{\max }}\_steps}-{n}_{obs\_steps}}{{n}_{{\rm{\max }}\_steps}-{n}_{shortest\_steps}},$$

*where n*_*max_steps*_ is the maximum number of steps found in each phase across all subjects, *n*_*shortest_steps*_ is the number of shortest steps to reach reward (i.e. number of steps in optimal path), *n*_*obs_steps*_ is the number of steps a subjects actually took. A score of 1 on both indices suggests that a subject used the shortest path on every trial, i.e., the subject was primarily displaying a map-based strategy. In contrast, a score of 0 indicates that a subject always used a suboptimal longer path.

***I***_***ROUTE***_. In both *I*_*PATH*_ and *I*_*STEPS*_, a score of 0 does not necessarily indicate that subjects simply followed previously established route to reach rewards. To address this issue, we also calculated the proportion of trials where subjects simply repeated previously learned routes. We defined this index as

3 $${I}_{ROUTE}=\frac{{n}_{route\_trial}}{{n}_{route\_trial}+{n}_{shortest\_trial}\,},$$

*where n*_*route_trial*_ is the number of trials when subjects repeated learned routes. The minimum overlap between the current path and the path in the encoding phase need to be at least 60% to be counted towards the index I_ROUTE_. By using the number of repetitions of a path as quantitative measure in our *I*_*ROUTE*_ index, we do not have to define an arbitrary single cutoff value for how often a path needs to be repeated in order to be considered as a well-known route. Note that for the encoding phase, this index might be less meaningful because subjects were still learning the environment and their task was to establish a certain route.

*I*_*PATH*_, *I*_*STEPS*_, and *I*_*ROUTE*_ were first calculated for each phase separately. To get a general idea on how participants navigate throughout the entire experiment, we then averaged each index over all three phases of the wayfinding task.

### Reinforcement learning model

We modelled the sequence of subjects’ choices (*a*_*i*_) by comparing them step by step to those predicted by different learning algorithms as having encountered the same state (*s*), action (*a*) and reward (*r*). As we had 5 by 5 grid, the wayfinding task consisted of 25 states and in each state, subjects could have up to three actions depending on which direction the subject was facing. The wayfinding task consisted of three rewards, the goal for both model-free and model-based algorithms is to learn the state-action value function *Q(s*,*a)* mapping at each state-action pair to each reward. We assume no interference or generalization between the three rewards conditions, and thus each algorithm was subdivided into three independent task set, one for each reward.

#### Model-free reinforcement learning

For model-free learning, we used the SARSA with eligibility traces (SARSA (λ)) to calculate model-free value or *Q*_*MF*_^89^. This algorithm has three free parameters: learning-rate (α), inverse temperature (β) and eligibility parameter (λ). Each state-action pair is associated with a value *Q*_*MF*_*(s*,*a)* all initially set to 0. The eligibility trace Z, set to 1 at the beginning of the trial and assumed not to be carried over from trial to trial, allows us to update each state-action pair along a subject’s encountered trajectory. For every trial *t* in which the subject located the reward (*r*), the state-action value is updated for each step *i* in that trial according to the following:

4 $$\,{Q}_{MF}({s}_{i,t+1},{a}_{i,t+1})\leftarrow {Q}_{MF}({s}_{i,t},{a}_{i,t})+\alpha \,{\delta }_{i,t}\,{Z}_{i},$$

where

5 $${\delta }_{i,t}\leftarrow R+{Q}_{MF}({s}_{i+1,t},{a}_{i+1,t})-{Q}_{MF}({s}_{i,t},{a}_{i,t}),$$

and

6 $$Z({s}_{i,t},{a}_{i,t})\leftarrow {\rm{\lambda }}\,Z({s}_{i,t},\,{a}_{i,t}).$$

#### Model-based reinforcement learning

A model-based approach learns the configuration of the grid world and computes action values by searching across possible trajectories to locate the reward^48^. Based on the grid-world configuration, we compute state-action values based on a planning process terminating at reward states. Specifically, for each action *a* in room *s*, we first initialized all *Q*_*MB*_*(s*,*a)* to 0. Then, for all state-action pairs *(s*,*a)* and adjacent (next room) state-action pairs (*s’*, *a’*) we iteratively perform the following:

7 $${Q}_{MB}(s,a)\leftarrow \{\begin{array}{cc}R(s\text{'}) & if\,R(s\text{'})\ne 0\\ ma{x}_{a^{\prime} \in A}{Q}_{MB}(s^{\prime} ,a^{\prime} )-(\gamma \ast ma{x}_{a^{\prime} \in A}{Q}_{MB}(s^{\prime} ,a^{\prime} )) & otherwise\end{array}$$

The algorithm has one fixed parameter γ that is set to 0.1. We took model-based values (*Q*_*MB*_) to be the values resulting after the algorithm converged (this occurred within 25 iterations). Note that computations of model-based value did not depend on the trial *t* or step *i* of the subject.

#### Hybrid model

In addition to model-free and model-based algorithm, we also considered a hybrid model^36,90^ in which the model predicted values for the actions are calculated as a weighted linear combination of the values from model-free and model-based algorithms:

8 $${Q}_{hybrid}=(1-\omega ){Q}_{MF}+\omega \,\,{Q}_{MB}$$

The relative degree that the model-based algorithm contributed over the model-free is captured by the weight parameter (ω). We took this ω as a free parameter, which was fitted individually for each subject but assumed to be constant throughout a single phase of the wayfinding task.

#### Model fitting

For each algorithm, we estimated a set of free parameters separately for each subject and for each phase of the wayfinding task by mean of hierarchical model fitting^84^. Further details on the model fitting and calculation of model evidence are provided in the SI Methods.

#### Analysis of reaction times (RT)

We computed, across subjects, correlation coefficients between subjects’ navigation indices and average RT in all choices of the subject. For the analysis of the relationship between distance to goal and RT we first regressed, separately for each subject, the distance to the reward for every choice on RT. We then took the resulting β-parameters of every subject into a second level analysis and tested against 0 with a t-test. This ensured that subjects were treated as random factor in the analysis. Note that we used only across subjects’ analysis in these tests (i.e. this way we could test if subjects who are on average more map-based showed on average higher RT) compared to analyzing individual trials within subjects. The reason for this is that our indices give a relative degree of the strategy for every subject over an entire phase of the experiment but do not classify individual trials into map or route trials.

### fMRI Data Analysis

#### General Linear Model (GLM) for fMRI data analysis

An event related analysis was applied on two levels using the general linear model approach as implemented in SPM12. Individual (random-effects) model parameters were used to generate regressors for the analysis of the fMRI data. The GLM included the following: event regressors covering the time when subjects saw (1) the instruction, (2) the room, (3) chose which direction they wanted to go (button press), (4) animation of movement, and (5) seeing the reward. Our analysis focused on the times when subject entered each room and the button press to indicate where to go next. For our primary hypothesis, the decision time points were parametrically modulated by (1) model-free chosen values (*Q*_*MF*_*(s*,*a))* and (2) model-based chosen values (*Q*_*MB*_*(s*,*a))*). We used separate regressors for each task phase, and the beta coefficients were subsequently averaged across phases at the contrast level. Parametric regressors were not serially orthogonalized, thus allowing each regressor to account independently for the response at each voxel. Using this approach, we let the model-free and model-based value regressor directly compete for variance in the BOLD signal. In this approach, only variance that is exclusively explained by one or the other regressor is assigned to the regressor but not the variance that is shared by both.

All regressors were convolved with the canonical hemodynamic response function as provided by SPM12 and its temporal derivative. The six rigid-body motion parameters from head motion correction were also included in the model as regressors of no interest. At the first level, linear weighted contrasts were used to identify effects of interest, providing contrast images for group effects analyzed at the second (random-effect) level.

#### Second level analysis

Calculated linear contrasts of parameter estimates, from the first level GLM analysis, for each regressor were then brought to the separate second level random-effects analysis, wherein one sample t-test provided effect for each regressor of interest. For correction of multiple comparisons, we set our significance threshold at *P < 0*.*05* whole-brain FWE corrected for multiple comparison at cluster level. The minimum spatial extent, k = 25, for the threshold was estimated based on the underlying voxel-wise *P* value with a cluster defining threshold of *P* = 0.001.

Details on the fMRI preprocessing are provided in the Supplementary Method.

### Region of Interest Analysis

We extracted data for all region of interest analyses using a cross-validation leave-one-out procedure: we re-estimated our second-level analysis 27 times, always leaving out one subject^91^. Starting at the peak voxel for the value signals in mPFC and right parahippocampal gyrus we selected the nearest maximum in these cross-validation second-level analyses. Using that new peak voxel, we then extracted the data from the left-out subject and averaged across voxels within a 4 mm sphere around that peak.

We then extracted ß-parameter estimates using either model-free or model-based parametric value regressors in these ROIs and calculate the correlation between these β and the fitted parameter ω from our behavioral hybrid learning model. This analysis provides additional information over the previous GLM analysis: the GLM identifies regions in which BOLD activity fluctuates with value signals of model-free and model-based RL on a population level without considering to which degree an individual subject of the group uses the corresponding strategy. The ROI analysis the tests the hypothesis that activity in these regions, which correlate with value signals, is indeed related to the degree at which an individual subject employs that strategy behaviorally.

Since circular analysis, the use of the same dataset and contrast for selection and selective analysis, has been a common pitfall in systems neuroscience^92^, we pay particular attention to avoid it here. Our ROI analysis is not subject to such ‘double-dipping’ for the following reasons: (1) we used a cross-validation leave-one-out procedure to define our regions of interest, ensuring that the data that is used to define the ROI is independent from the data extracted of this ROI (2) the value-contrasts used in defining and extracting the ROI data (ß) are from a different RL model then the one providing the behavioral ω parameter that is correlated against the ROI data, making both independent by design (see description above).

### Data Availability and Resource Sharing

The datasets generated during the current study are available from the first author Dian Anggraini (dian.anggraini.lmu@outlook.de) upon reasonable request. The following software were used for the experimental design, VR environment for the wayfinding task, and data analysis: Worldviz Vizard 4.0 (https://worldviz.com); SPM12 (https://fil.ion.ucl.ac.uk, RRID:SCR_007037); Xjview (https://alivelearn.net/xjview/, RRID:SCR_008642); SPM Anatomy Toolbox (https://fz-juelich.de/, RRID:SCR_013273); mricron (https://nitrc.org/mricron, RRID: SCR_002403).

## Electronic supplementary material

Supplementary Information
