# Supplementary material for: Neural signatures of reinforcement learning correlate with strategy adoption during spatial navigation
Source: Sci Rep. 2018 Jul 4;8:10110. doi: 10.1038/s41598-018-28241-z (PMC6031619; doi:10.1038/s41598-018-28241-z)
Supplement: Supplementary file 1 — Supplementary Information [file 41598_2018_28241_MOESM1_ESM.docx]

**Supplementary Information**

**Neural signatures of reinforcement learning correlate with strategy adoption during spatial navigation**

Dian Anggraini^1,4^, Stefan Glasauer^2,3,4^, and Klaus Wunderlich^1,3,4*^

^1^Department of Psychology; Ludwig-Maximilians-Universitaet Muenchen; Munich 80802; Germany.

^2^Center for Sensorimotor Research; Department of Neurology; Ludwig-Maximilians-Universitaet Muenchen Klinikum Grosshadern; Munich 81377; Germany.

^3^Bernstein Center for Computational Neuroscience Munich; Planegg-Martinsried 82152; Germany.

^4^Graduate School of Systemic Neuroscience LMU Munich; Planegg-Martinsried 82152; Germany.

*To whom correspondence should be addressed:

Klaus Wunderlich, Department of Psychology Ludwig-Maximilians-Universitaet Muenchen. Leopold Str. 13 80802 Munich, Germany. Email: Klaus.Wunderlich@lmu.de

**Supplementary Results and Discussion**

**Frequency of visited rooms and directions**

For each phase of the experiment we analyzed the frequencies of rooms visited across all subjects. In all phases, starting locations and reward rooms were visited slightly more frequently than the other rooms (Figure S2).

We also analyzed frequencies of chosen direction (left, center, right). We found a main effect for direction only in the encoding phase (*F_(78)_* = 3.53, *P* = 0.03), where subjects had a slight preference to go left or straight compared to going right. This effect was no longer present in retrieval (*F_(78)_* = 0.049, *P* = 0.95) and search phases (*F_(78)_* = 0.34, *P* = 0.71).

**Changes in Strategy Adoption across Three Different Phase of the Wayfinding Task**

To test whether there is a significant change in strategy adoption across the three different phases of the wayfinding task, we run a repeated measure ANOVA for each navigation indices and the fitted parameter ω with phase as within subject factor. We found that there is a main effect of phase for *I_PATH_* (*F(_2,78)_* = 10.49, *P* = 0.001), *I_STEPS_* (*F_(2,78)_* = 5.7989, *P* = 0.004), and *I_ROUTE_* (*F_(2,78)_* = 27.717, *P* = 1.18 × 10^-9^). Similarly, we also found that there is a significant main effect of phase (*F_(2,78)_* = 12.86, *P* = 1.67 × 10^-5^) for the fitted parameter ω.

As illustrated in Supplementary Figure S3, participants tend to use on average more optimal paths (and less extra steps) to reach rewards during the retrieval and search phases in comparison with the encoding phase. Similarly, participants repeated fewer paths in the search phase as compared to the encoding and retrieval phase. There was a tendency to repeat paths in the retrieval phase (slightly higher *I_ROUTE_*) because participants started from the same starting position as during encoding. We expected this trend because our encoding phase was designed to encourage participants to repeat their paths from the starting position to reward 1, then to reward 2, then to reward 3 and being in the same starting position would serve as a stimulus to trigger the route-bases response. In contrast, during the search phase, started from novel locations, prompting to plan optimal paths. Note that random exploration of the maze during the encoding phase might have also affected the index values. Changes in ω across the three phases also indicate that participants shifted towards more model-based choices as they went from encoding phase to retrieval phase and to search phase.

**Strategy Adoption in Three Different Phases of the Wayfinding Task**

To test if our RL algorithm captures the variability in strategy adoption during navigation we calculated correlation coefficients of both indices (*I_PATH_* and *I_STEPS_*) with the fitted weight (ω) for each phase of the wayfinding task (Fig. S4).

***Encoding phase.*** Significant correlation between ω and *I_PATH_* (r = 0.84, two-tailed t-test *P* < 5.8 x 10^-8^), *I_STEPS_* (r = 0.87, two-tailed t-test *P* < 7.6 x 10^-9^), as well as *I_ROUTE_* (r = -0.75, two-tailed t-test *P* < 8.7 x 10^-6^).

***Retrieval phase.*** Significant correlation between ω and *I_PATH_* (r = 0.73, two-tailed t-test *P* < 1.4 x 10^-5^), *I_STEPS_* (r = 0.52, two-tailed t-test *P* < 0.006), as well as *I_ROUTE_* (r = -0.65, two-tailed t-test *P* < 2.6 x 10^-4^).

***Search phase.*** Significant correlation between ω and *I_PATH_* (r = 0.56, two-tailed t-test *P* < 0.003), *I_STEPS_* (r = 0.40, two-tailed t-test *P* < 0.03), as well as *I_ROUTE_* (r = -0.45, two-tailed t-test *P* < 0.02). For aggregated data over all experimental phases see main text.

**Additional Behavioural Experiment without Monetary Reward**

To rule out the influence of an external monetary reward on our results, sixteen healthy female participants (19 – 29 years of age) took part in our additional behavioural experiment. The experimental procedure was almost the same as the main experiment, except subjects completed 12 trials (instead of as many as possible within a certain time) during the search phase. For this additional behavioural experiment, no reward is associated with target locations. In addition, subjects received a flat participation credit instead of monetary rewards for their participation.

We compared the fitted parameters from this behavioural experiment to the ones from the fMRI experiment (Fig. S5). Comparing the fitted parameters from the additional experiment (without reward) and the original experiment (with reward) using an independent-samples t-test, we found no significant differences for all parameter values. Model free (α: t(41) = 0.44, *P* > 0.66; λ: t(41) = 0.61, *P* > 0.54; β: t(41) = -0.78, *P* > 0.430), model-based (β: t(41) = 0.52, *P* > 0.60), and hybrid model (α: t(41) = 0.79, *P* > 0.22; λ: t(41) = -0.58, *P* > 0.56; β: t(41) = 1.72, *P* > 0.09; and ω: t(41) = -1.62, *P* = 0.11). In addition, we also replicate the correlation between parameter weight (ω) from the hybrid model and navigation indices. For the encoding phase, we found significant positive correlation between ω and *I_PATH_* (r = 0.54, two-tailed t-test *P* < 0.03) and *I_STEPS_* (r = 0.61, two-tailed t-test *P* < 0.01). For the retrieval phase, we found significant correlation between ω and *I_PATH_* (r = 0.91, two-tailed t-test *P* < 4.7 x 10^-7^), *I_STEPS_* (r = 0.60, two-tailed t-test *P* < 0.01), as well as *I_ROUTE_* (r = -0.822, two-tailed t-test *P* < 9.3 x 10^-5^). We also found significant correlation between ω and *I_PATH_* (r =0.83, two-tailed t-test *P* < 6.6 x 10^-5^), *I_STEPS_* (r = 0.72, two-tailed t-test *P* < 0.001), as well as *I_ROUTE_* (r = -0.78, two-tailed t-test *P* < 2.7 x 10^-4^) for the search phase.

**Additional exploratory hypotheses tested in the fMRI analysis**

We tested a few exploratory hypotheses, in addition to the results presented in the main text, and mention the results here for completeness.

***fMRI activity at time of entering the room***

There are several cognitive processes that subjects are faced with when entering each room, such as recognition of the objects, recall and matching to previously encountered rooms, identifying the location of the room in a cognitive map, calculating a navigation strategy and finally deriving an action choice from that. A lot of these processes have to take place before any decision can be made. Since our 3D environment is visually much more complex than a decision experiment where simple stimuli are presented, we expected the time of the response to be a better predictor than entering the room for when the brain computes navigation decisions. We therefore hypothesized that value signals would be represented strongest at the time of making a choice, which is the time information that we used in our GLM. However, we alternatively explored if there is any BOLD activity pertaining to model-free or model-based values already when subjects entered the room, i.e. preceding the actual decision point. From this analysis, we found neural correlates of model-free values in the right precuneus [x = 3, y = -70, z = 38] and of model-based values in right middle occipital gyrus [x = 39, y = -79, z = 14] but as expected those relationships were much weaker than when they are related to the time of the choice.

We also tested if the brain tracks the frequency of entering the current room, frequency of entering the current room and choosing the same direction, time since last entering the room, as well as whether the current room was located in the centre or the peripheral of the grid-world. We did not find any BOLD activity that are significantly correlated with these variables.

***Covariates at fMRI group level***

We tested the number of trials and MRT scores as covariates in our 2^nd^ level GLM analysis. There was no significant correlation between these covariates and BOLD signal anywhere in the brain that survived correction for multiple comparisons.

**Supplementary Methods**

**Wayfinding Task**

We built two 3D virtual environments with a commercially available software that displays a first person point of view in a fully textured grid world (Vizard version 4.0; WorldViz, LLC https://worldviz.com). The 3D environment consisted of a 5 by 5 grid of rooms. Every room contained distinct furniture and objects as landmarks to distinguish individual rooms. Three rooms were designated as reward rooms. Each reward room contained one of three objects that subjects had to find while navigating through the maze. For the practice session, conducted three to five days before the fMRI scanning sessions, subject practiced on a smaller 4 by 4 grid world with different room furnishings but otherwise similar to the main task. Subjects could move from one room to another by pressing keys on a button box. Subjects could move forward, left or right. Backtracking was not allowed. Following their response, subjects viewed an animated movement sequence of the selected direction. It is important to note that a wayfinding task where participants are required to choose a direction and are then moved through the environment based on their directional choices is well established in the field of spatial navigation (for more examples see ^1-6^).

After entering the MRI, we instructed subjects to freely explore the 5 by 5 grid, without any rewards present, to learn to move with the button press and to get comfortable with the stimulus materials. Subjects had 35 choices to explore the setting which gave them enough time to explore all possible states of the environment, i.e. all the rooms, during this initial exploration. The following task consisted of three phases: (1) encoding, (2) retrieval, and (3) search. During the encoding phase, subjects always started in the same starting position and were asked to collect three rewards in a specific order over eight trials. During the retrieval phase, subjects were instructed to collect one specific reward at a time from the same starting position as in the encoding phase. Rewards were positioned at the same location as in encoding phase and selected in random order. Subject completed 15 trials in the retrieval phase. During the search phase, subjects had to collect a specific randomly chosen reward, each time starting from a different starting position. Subjects had 20 minutes to do as many trials as possible. Note that using short routes would allow subjects to reach target rooms more quickly and hence collect more rewards. After the experiment subjects a part of subjects’ financial compensation was related to the number of collected rewards. Use of a map-based strategy in this phase was therefore beneficial to subjects’ payment.

**Mental Rotation Task**

The Vandenberg and Kuse Mental Rotation Test (MRT) is comprised of 24 items, six items on four separate pages in the test booklet ^7^. Each item is comprised of a row of five line drawings including a geometrical target figure in the left-most position followed by four response-choice figures: two rotated reproductions of the target and two distractors. The subject’s task is to indicate which two of the four response choice figures are rotated reproductions of the target figure. In each item there are always two and only two correct figures and two incorrect distractor figures. For each item, subjects were instructed to find two response choices with figures identical to the target figures. Subjects had 10 minutes to complete the task and were informed when there were 5 minutes remaining and again when there were 2 minutes remaining. Instructions emphasized that subjects should refrain from guessing. Score of 1 is given for every correct answer. Thus, the maximum score of the MRT is 48 ^8^.

**Magnetic Resonance Imaging (MRI) Images Acquisition**

We performed functional imaging using a 3T whole-body Siemens MAGNETOM Verio scanner with an 8-channel head coil located at the Klinikum der Universität München. T2* echo-planar images were obtained with 2390 ms repetition time (TR) with an acquisition matrix of 64 x 64, an echo time (TE) of 30 ms, a flip angle of 90^0^, and field of view of 192 x 192 mm. Each volume consisted of 30 (3.0 x 3.0 x 3.0 mm^3^ voxels) axial slices with 15% gap. We completed five dummy scans at the beginning of each run to allow for stabilization of the MR signal. In addition, a structural whole brain scan was acquired using an MP-RAGE T1-weighted sequence (TR/TE = 11.0 / 4.76 ms) with 256 x 256 x 160 acquisition matrix, 1 x 1 x 1 mm3 voxel size, 15^0^ flip angle, and a field of view of 256 x 256 mm.

**Model Fitting**

For each algorithm, we calculated a probability of choice based on the following softmax decision rule (Luce choice rule):

$p_{t}= \frac{\exp\beta Q_{t}(s_{t}, a)}{\sum_{a^{'}\in A} \exp\beta Q_{t}(s_{t}, a')}$ [S1]

The parameter β is the inverse temperature representing the degree of stochasticity of subjects’ action selection.

We estimated a set of free parameters (θ) for each algorithm separately for each subject by mean of hierarchical model fitting ^9^. This approach allows us to model the data generation explicitly, i.e. to incorporate a model how parameters vary across the population. This means, when we recruited a subject *i* from population, we also draw a set of parameters according to some statistical distribution that characterize the distribution of parameters in the population. The first step in hierarchical model fitting is applying logistic (α, ω, λ) and exponential (β) transformation before fitting parameters. This step transforms bounded parameter into Gaussian distributed parameter values with population mean and standard deviation. In the equation below, the Greek alphabets represent the parameters we used in the model, while the Latin alphabets stand for parameters in the logistic transformations that range from -∞ to ∞.

$\alpha=\frac{1}{1+exp(-a)}$; $\omega=\frac{1}{1+exp(-w)}$; λ$=\frac{1}{1+exp(-l)}$; $\beta=exp(b)$ [S2]

Adopting a model of the parameters in the population gives us a two-level hierarchical model of how a full dataset is produced. This means, each subjects’ parameters are drawn from population distributions, then the Q values and the observable choice are generated, according to an RL model with those parameters. The full equation that relates these population-level parameters to a particular subject’s choice, ***c_i_***, is then the probability given to them by the parameterized model averaged over all possible settings of the individual subject’s parameters according to their population distribution:

𝐿 = 𝑃(𝑐_𝑖_|𝜇_𝑎_, 𝜇_𝑙_, 𝜇_𝑏_, 𝜇_𝑤_, 𝜎_𝑎_, 𝜎_𝑙_, 𝜎_𝑏_, 𝜎_𝑤_) =

∫ *P*(𝑐_𝑖_|𝑎_𝑖_, 𝑙_𝑖_, 𝑏_𝑖_, 𝑤_𝑖_)*P*(𝑎_𝑖_|𝜇_𝑎_, 𝜎_𝑎_)𝑃(𝑙_𝑖_|𝜇_𝑙_, 𝜎_𝑙_)𝑃(𝑏_𝑖_|𝜇_𝑏_, 𝜎_𝑏_) 𝑃(𝑤_𝑖_|𝜇_𝑤_, 𝜎_𝑤_) 𝑑𝑎_𝑖_𝑑𝑙_𝑖_𝑑𝑏_𝑖_𝑑𝑤_𝑖_ [S3]

We later estimated mean and variance of the parameter distribution in the population based on our subject sample. As an example, for parameter α:

$\mu_{a}= \frac{1}{N} \sum_{i}^{N} a_{i} and \sigma_{a}= \sqrt{\frac{1}{N} \sum_{i}^{N} \left( a_{i}- \mu_{a} \right)^{2}}$ [S4]

Following mean and variance estimation, we refitted single subject parameter values by minimizing over both the negative log likelihood of subjects’ choice given the parameters and the negative log likelihood for individual subject parameter values given the distribution of parameters in the population:

𝑃(𝑎_𝑖_, 𝑙_𝑖_, 𝑏_𝑖_, 𝑤_𝑖_|𝑐_𝑖_, 𝜇_𝑎_, 𝜇_𝑙_, 𝜇_𝑏_, 𝜇_𝑤_, 𝜎_𝑎_𝜎_𝑙_, 𝜎_𝑏_, 𝜎_𝑤_)

∝ *P*(𝑐_𝑖_|𝑎_𝑖_𝑙_𝑖_𝑏_𝑖_𝑤_𝑖_) × *P*(𝑎_𝑖_, 𝑙_𝑖_, 𝑏_𝑖_, 𝑤_𝑖_|𝜇_𝑎_, 𝜇_𝑙_, 𝜇_𝑏_, 𝜇_𝑤_, 𝜎_𝑎_𝜎_𝑙_, 𝜎_𝑏_, 𝜎_𝑤_) [S5]

As we refitted the subjects’ parameter by minimizing the negative log likelihood (minimization using simulated annealing as implemented in the MATLAB Global Optimization Toolbox), then the estimated parameters were drawn toward the group means. This reflects the fact that the data for other subjects in a population are also relevant to estimating a subject’s parameters. Thus, in the context of hierarchical model, our approach (combination of prior and likelihood in Bayes rule) specifies how to balance population information with data about the individual in estimating our subjects’ parameters.

***Model evidence.*** To approximate the model evidence, we computed the Bayesian Information criterion (BIC) as follows:

$l\left( \hat{\theta} \right)+ \frac{m}{2}\log n$ [S6]

where 𝑙(𝜃̂) is the negative log-likelihood of data at the maximum likelihood parameters θ; *m* is the number of free parameters optimized; and *n* is the number of choices the subject made. The BIC was calculated based on the hierarchical model fitting. Note that since subjects might employ either model-free or model-based strategy in individual trials, we did not choose RL model based on aggregated BIC of the subjects.

**fMRI Data Analysis**

Neuroimaging data were processed and analysed using SPM12 toolbox (www.fil.ion.ucl.ac.uk/spm/software/spm12/). An event-related statistical analysis was applied to the images on two levels using the general linear model approach as implemented in SPM12.

***Preprocessing of fMRI data.*** Functional images were realigned for head motion and coregistered between runs and to the structural images. The images were then spatially normalized to Montreal Neurological Institute (MNI) space using the normalization parameters generated during the segmentation for each subject’s anatomical T1 scan and resampled to 2 mm isotropic voxels. Subsequently, all images were smoothed with an 8 mm full width at half maximum Gaussian kernel.

**Supplementary Figures**

**Figure S1.** **Example of five representative subjects’ paths in encoding and search phases** During the encoding phase, most subjects started by exploring the environment. They then either (**A**) established a certain route to go from one reward to the next or (**B**) found the shortest paths to go between one reward and the next. During retrieval and search phases some subjects mostly used the (**C**) shortest path to retrieve rewards while others either (**D**) took a detour before reaching the reward or (**E**) used the route they established during the earlier encoding phase. No subject chose exclusively one strategy over the other. Note that starting direction was always facing upward and subjects could not go backwards, so that from starting position **S** the downward path was not permitted.


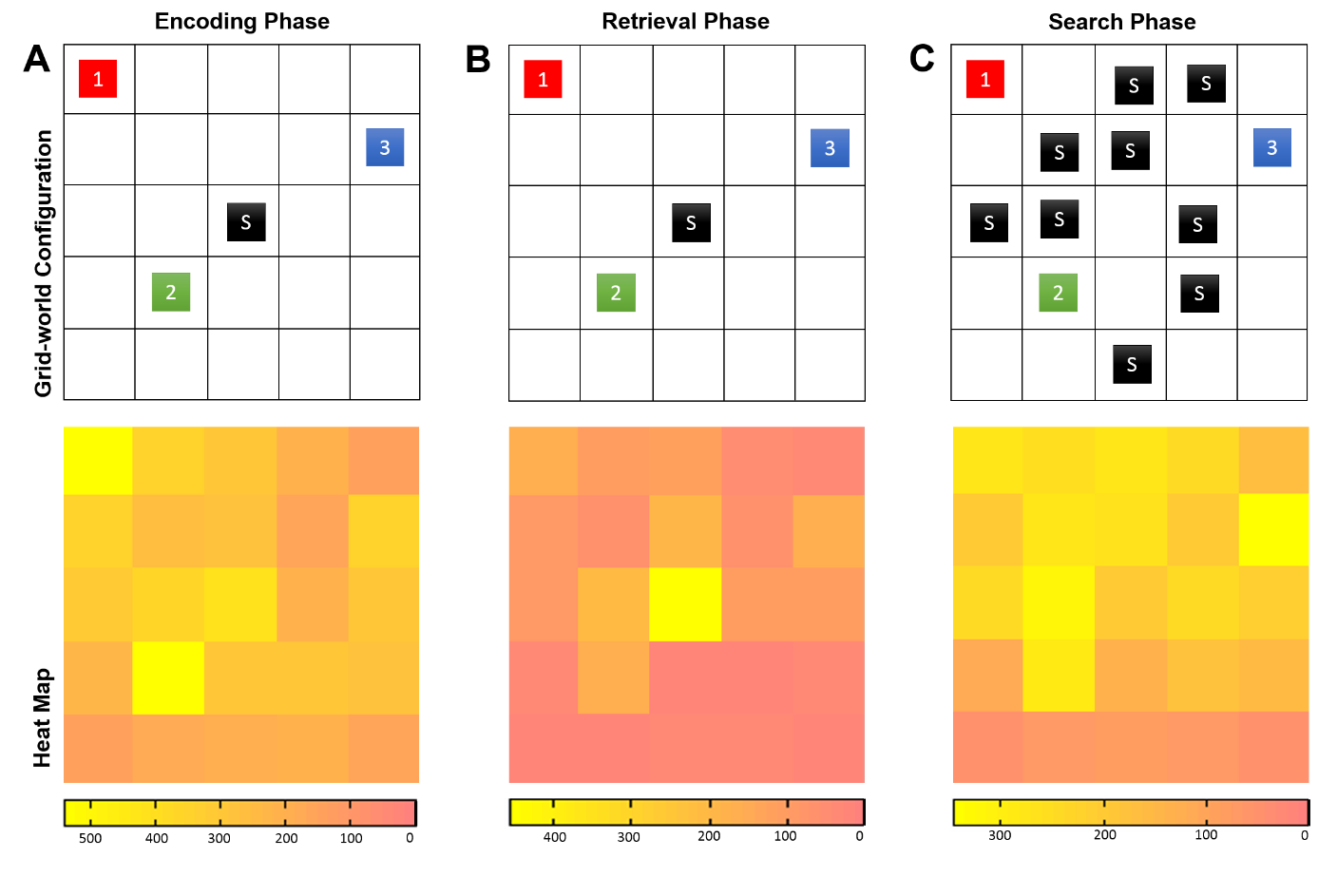


**Figure S2.** **Heat map showing frequency of visited rooms across all participants**

(**A**) During the encoding phase, the starting position (the room in the middle of the maze) along with reward 1, reward 2, and reward 3 were visited most frequently. Reward rooms 1 and 2 were visited slightly more frequently than reward room 3. An explanation for this is that in the beginning, while subjects were still learning the layout of the maze, some subjects revisited the rooms of early rewards on their search for later rewards.

(**B**) During the retrieval phase, subjects also always started from the same starting position (center of the maze), which was therefore frequented most often.

(**C**) During the search phase, subjects started from multiple possible starting positions.

**Figure S3. Navigation indices for encoding, retrieval, and search phase**

(**A**) Subjects used more optimal paths to reach rewards during search phase compared to the encoding phase (*t_(26)_* = 7.384, *P* = 4.89 x 10^-8^).

(**B**) During the search phase, subjects also used less extra steps in comparison to the retrieval phase (*t_(26)_* = -5.45, *P* = 6.64 x 10^-6^).

(**C**) Similarly, participants repeated fewer paths in the search phase as compared to the retrieval phase (*t_(26)_* = -11.87, *P* = 7.74 x 10^-12^) and encoding phase (*t_(26)_* = -6.41, *P* = 6.25 x 10^-7^).

(**D**) An increase in ω across the three phases indicate that participants shifted towards more model-based choices along the experiment. Subjects integrate more model-based during the search phase compare to retrieval phase (*t_(26)_* = 4.786, *P* = 3.57 x 10^-5^) and encoding phase (*t_(26)_* = 9.327, *P* = 9.39 x 10^-10^).

Asterisks indicate significant differences in the post-hoc t-test.


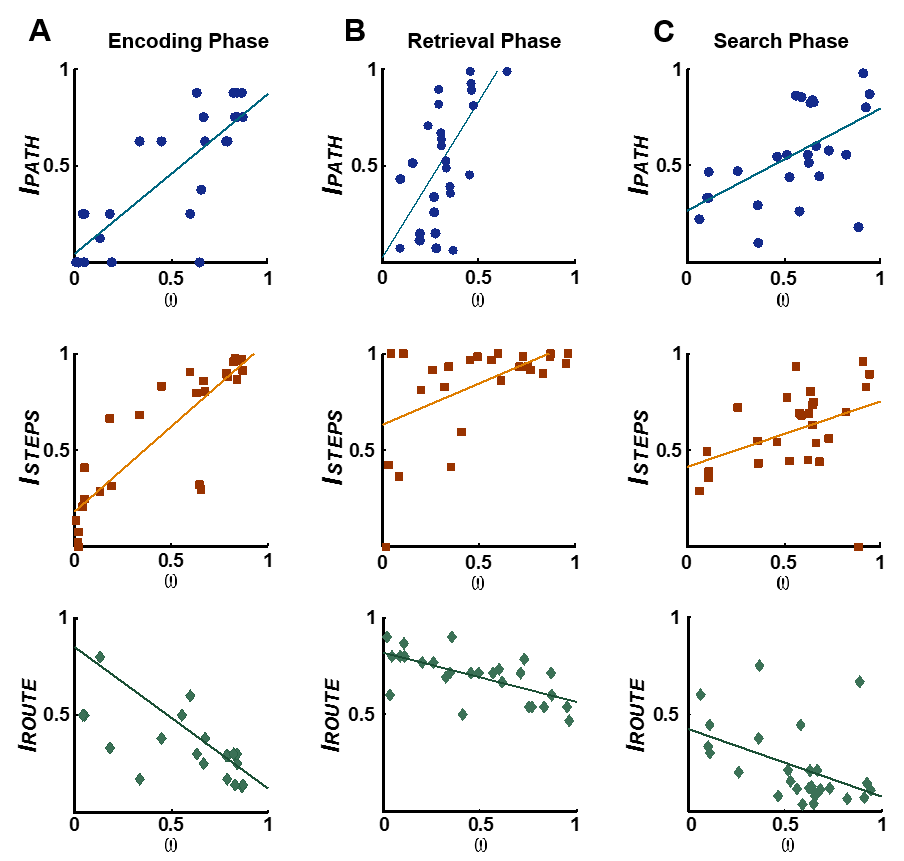


**Figure S4. Correlation of navigation indices (*I_PATH_*, *I_STEPS,_ and I_ROUTE_* ) with parameter weight (ω) in every phase**

(**A**) For the encoding phase, significant correlation between ω and *I_PATH_* (r = 0.84, two-tailed t-test *P* < 5.8 x 10^-8^), *I_STEPS_* (r = 0.87, two-tailed t-test *P* < 7.6 x 10^-9^), as well as *I_ROUTE_* (r = -0.75, two-tailed t-test *P* < 8.7 x 10^-6^).

(**B**) For the retrieval phase, significant correlation between ω and *I_PATH_* (r = 0.73, two-tailed t-test *P* < 1.4 x 10^-5^), *I_STEPS_* (r = 0.52, two-tailed t-test *P* < 0.006), as well as *I_ROUTE_* (r = -0.65, two-tailed t-test *P* < 2.6 x 10^-4^).

(**C**) For the search phase, significant correlation between ω and *I_PATH_* (r = 0.56, two-tailed t-test *P* < 0.003), *I_STEPS_* (r = 0.40, two-tailed t-test *P* < 0.03), as well as *I_ROUTE_* (r = -0.45, two-tailed t-test *P* < 0.02).

These correlations confirm that RL algorithms capture the variability in strategy adoption during navigation.

**Figure S5. Distribution of fitted parameters for the behavioural and fMRI experiment as well as correlation of navigation indices (*I_PATH_*, *I_STEPS,_ and I_ROUTE_*) with weight on every phase of the wayfinding task**

(**A**) Distribution of fitted parameters from the additional behavioural experiment and the fMRI experiment for α, λ, and β from model free and parameter β from model-based. We found no significant difference between fitted parameters from the behavioural and fMRI experiment for model-free (α: *P* > 0.660; λ: *P* > 0.546; β: *P* > 0.430), model-based (β: *P* > 0.60).

(**B**) Distribution of fitted parameters from the additional behavioural experiment and the fMRI experiment for α, λ, β, and ω from hybrid model. We found no significant difference between fitted parameters from the behavioural and fMRI experiment for hybrid model (α: *P* > 0.216; λ: *P* > 0.560; β: *P* > 0.067; and ω: *P* > 0.12).

(**C**) For the encoding phase, significant correlation between ω and *I_PATH_* (r = 0.54, two-tailed t-test *P* < 0.03) and *I_STEPS_* (r = 0.61, two-tailed t-test *P* < 0.01). (*D*) For the retrieval phase, significant correlation between ω and *I_PATH_* (r = 0.91, two-tailed t-test *P* < 4.7 x 10^-7^), *I_STEPS_* (r = 0.60, two-tailed t-test *P* < 0.01), as well as *I_ROUTE_* (r = -0.822, two-tailed t-test *P* < 9.3 x 10^-5^). (*E*) For the search phase, significant correlation between ω and *I_PATH_* (r =0.83, two-tailed t-test *P* < 6.6 x 10^-5^), *I_STEPS_* (r = 0.72, two-tailed t-test *P* < 0.001), as well as *I_ROUTE_* (r = -0.78, two-tailed t-test *P* < 2.7 x 10^-4^).


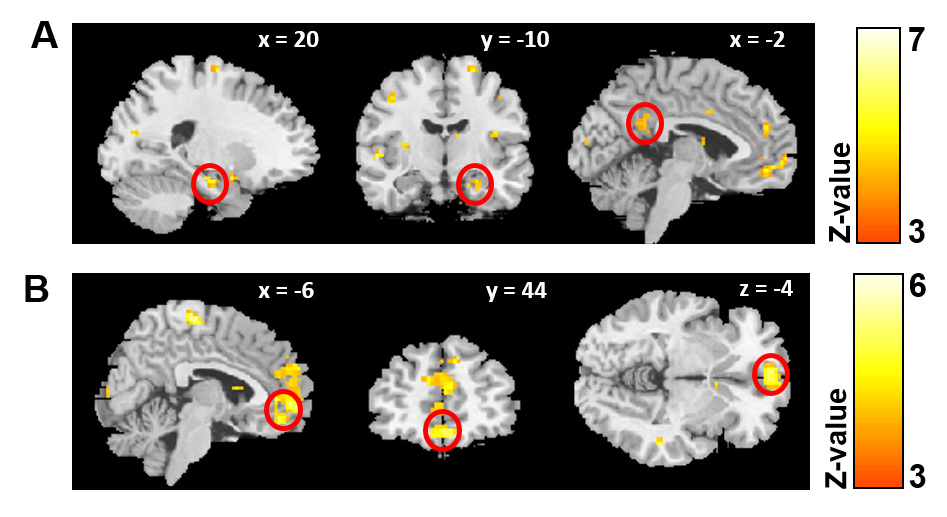


**Figure S6.** **Distance and model-based or model-free regressor in one GLM**

(**A**) Neural correlates of model-based action values in parahippocampal/hippocampal region [x = 21, y = -13, z = -19] and retrosplenial cortex [x = -6, y = -43, z = 17]. Even when we put model-based and distance regressors as non-orthogonalized parametric modulators in the same GLM, we still see the correlated BOLD activity to model-based valuations. Note that the model-based algorithm computed value regressors using both subject’s state (i.e. location relative to goal) and chosen action. Consequently, for some trials, model-based regressors did not necessarily reflect the optimal value of the subject’s state. These findings rule out that the BOLD correlations with model-based value signals are only a spurious correlate of the distance to the reward.

(**B**) Neural correlates of model-free action values in the medial prefrontal cortex. Similar to model-based value signals, even after including the shortest distance regressor, we still found significant correlate of model-free action values along the left medial prefrontal cortex [x = -3, y = 44, z = -13] extending to the bilateral superior medial gyrus [x = 6, y = -59, z = 20 and x = -12. Y = -47, z = 23]. This indicates that the results are indeed correlates of model-free value signals and that the shortest distance is not a confounding factor of the mPFC activity.

**Supplementary Tables**

# Table S1. Map/Route Indices, Fitted Parameter Weight (ω) and Mental Rotation Task (MRT) score

| **Subject** | ***I_PATH_*** | ***I_STEPS_*** | ***I_ROUTE_*** | **Weight** | | | | **Aggregate BIC** | | | **MRT** |
| --- | --- | --- | --- | --- | --- | --- | --- | --- | --- | --- | --- |
|  |  |  |  | **Encoding** | **Retrieval** | **Search** | **Average** | **MF** | **MB** | **Hybrid** |  |
| 1 | 0.12 | 0.36 | 0.80 | 0.19 | 0.09 | 0.06 | 0.11 | 321.2 | 359.3 | 337.1 | 14 |
| 2 | 0.46 | 0.84 | 0.38 | 0.18 | 0.34 | 0.56 | 0.36 | 205.0 | 206.0 | 214.5 | 33 |
| 3 | 0.35 | 0.65 | 0.49 | 0.05 | 0.73 | 0.67 | 0.48 | 319.7 | 321.1 | 329.6 | 31 |
| 4 | 0.06 | 0.15 | 0.88 | 0.02 | 0.02 | 0.37 | 0.13 | 520.9 | 543.3 | 536.4 | 7 |
| 5 | 0.76 | 0.96 | 0.23 | 0.86 | 0.96 | 0.94 | 0.92 | 198.8 | 130.3 | 168.6 | 36 |
| 6 | 0.48 | 0.88 | 0.43 | 0.60 | 0.77 | 0.92 | 0.76 | 228.7 | 175.6 | 214.2 | 18 |
| 7 | 0.31 | 0.42 | 0.53 | 0.13 | 0.41 | 0.11 | 0.22 | 445.7 | 440.0 | 457.6 | 9 |
| 8 | 0.62 | 0.83 | 0.33 | 0.84 | 0.71 | 0.59 | 0.71 | 173.1 | 176.4 | 174.5 | 17 |
| 9 | 0.58 | 0.83 | 0.37 | 0.67 | 0.57 | 0.65 | 0.63 | 219.4 | 204.3 | 229.0 | 37 |
| 10 | 0.45 | 0.63 | 0.44 | 0.55 | 0.53 | 0.78 | 0.61 | 223.0 | 206.9 | 227.7 | 23 |
| 11 | 0.53 | 0.75 | 0.32 | 0.79 | 0.75 | 0.62 | 0.72 | 260.8 | 227.3 | 255.9 | 21 |
| 12 | 0.49 | 0.77 | 0.36 | 0.67 | 0.60 | 0.68 | 0.65 | 242.4 | 211.5 | 238.8 | 36 |
| 13 | 0.58 | 0.81 | 0.26 | 0.87 | 0.95 | 0.73 | 0.85 | 260.5 | 219.0 | 247.3 | 26 |
| 14 | 0.25 | 0.67 | 0.64 | 0.64 | 0.45 | 0.26 | 0.45 | 337.6 | 332.7 | 349.3 | 19 |
| 15 | 0.55 | 0.90 | 0.41 | 0.78 | 0.05 | 0.63 | 0.49 | 170.0 | 157.8 | 167.3 | 32 |
| 16 | 0.15 | 0.28 | 0.78 | 0.02 | 0.32 | 0.89 | 0.41 | 527.1 | 522.4 | 546.1 | 16 |
| 17 | 0.33 | 0.53 | 0.46 | 0.04 | 0.83 | 0.10 | 0.33 | 407.1 | 393.6 | 417.9 | 31 |
| 18 | 0.48 | 0.80 | 0.44 | 0.84 | 0.11 | 0.53 | 0.49 | 189.1 | 178.3 | 194.2 | 36 |
| 19 | 0.18 | 0.42 | 0.68 | 0.01 | 0.03 | 0.58 | 0.21 | 497.7 | 499.9 | 513.6 | 20 |
| 20 | 0.55 | 0.82 | 0.34 | 0.79 | 0.20 | 0.66 | 0.55 | 225.6 | 214.1 | 223.5 | 35 |
| 21 | 0.48 | 0.75 | 0.29 | 0.34 | 0.61 | 0.82 | 0.59 | 281.3 | 259.3 | 280.1 | 33 |
| 22 | 0.46 | 0.79 | 0.42 | 0.45 | 0.11 | 0.47 | 0.34 | 220.5 | 215.5 | 226.1 | 34 |
| 23 | 0.71 | 0.97 | 0.36 | 0.82 | 0.87 | 0.91 | 0.87 | 175.2 | 115.6 | 153.8 | 38 |
| 24 | 0.16 | 0.57 | 0.72 | 0.05 | 0.26 | 0.37 | 0.23 | 399.8 | 393.5 | 413.8 | 11 |
| 25 | 0.13 | 0.28 | 0.78 | 0.02 | 0.35 | 0.11 | 0.16 | 310.5 | 398.0 | 326.8 | 18 |
| 26 | 0.60 | 0.83 | 0.37 | 0.63 | 0.87 | 0.63 | 0.71 | 223.2 | 202.3 | 220.6 | 34 |
| 27 | 0.52 | 0.91 | 0.35 | 0.83 | 0.49 | 0.52 | 0.61 | 191.7 | 156.7 | 183.5 | 27 |

**Table S2. Distribution of subjects’ individual maximum likelihoods and parameter estimates**

| **Model-free** | | | | **Model-based** | | |  | **Hybrid** |  |  | **Random** |  |
| --- | --- | --- | --- | --- | --- | --- | --- | --- | --- | --- | --- | --- |
| **Encoding Phase** | |  |  |  |  |  |  |  |  |  |  |  |
| β | 5.70 | **11.06** | 13.43 | 3.01 | **10.39** | 19.54 | 11.61 | **13.93** | 17.67 |  |  |  |
| γ |  |  |  | - | **0.1** | - | - | **0.1** | - |  |  |  |
| α | 0.08 | **0.36** | 0.62 |  |  |  | 0.09 | **0.23** | 0.76 |  |  |  |
| λ | 0.67 | **0.78** | 0.90 |  |  |  | 0.74 | **0.89** | 0.95 |  |  |  |
| ω |  |  |  |  |  |  | 0.08 | **0.59** | 0.78 |  |  |  |
| NLL | 79.13 | **95.63** | 210.32 | 68.81 | **94.42** | 232.85 | 68.04 | **91.53** | 210.22 | 116.29 | **129.74** | 238.42 |
| BIC | 86.39 | **103.19** | 218.78 | 71.26 | **96.94** | 235.69 | 78.86 | **138.24** | 222.52 | 116.29 | **129.74** | 238.42 |
|  |  |  |  |  |  |  |  |  |  |  |  |  |
| **Retrieval Phase** | |  |  |  |  |  |  |  |  |  |  |  |
| β | 5.08 | **6.16** | 9.20 | 9.79 | **13.49** | 19.06 | 8.63 | **11.17** | 15.34 |  |  |  |
| γ |  |  |  | - | **0.1** | - | - | **0.1** | - |  |  |  |
| α | 0.32 | **0.89** | 0.96 |  |  |  | 0.58 | **0.97** | 0.99 |  |  |  |
| λ | 0.55 | **0.88** | 0.94 |  |  |  | 0.41 | **0.83** | 0.97 |  |  |  |
| ω |  |  |  |  |  |  | 0.22 | **0.49** | 0.75 |  |  |  |
| NLL | 20.15 | **27.25** | 43.21 | 17.43 | **27.20** | 41.82 | 17.23 | **27.03** | 41.24 | 51.39 | **55.44** | 65.70 |
| BIC | 25.92 | **33.20** | 49.31 | 19.36 | **29.19** | 43.91 | 25.93 | **35.97** | 50.41 | 51.39 | **55.44** | 65.70 |
|  |  |  |  |  |  |  |  |  |  |  |  |  |
| **Search Phase** | |  |  |  |  |  |  |  |  |  |  |  |
| β | 1.89 | **2.49** | 3.53 | 3.43 | **6.55** | 9.05 | 2.89 | **5.05** | 7.15 |  |  |  |
| γ |  |  |  |  |  |  | - | **0.1** | - |  |  |  |
| α | 0.38 | **0.87** | 0.97 |  |  |  | 0.56 | **0.87** | 0.97 |  |  |  |
| λ | 0.6 | **0.86** | 0.86 |  |  |  | 0.63 | **0.86** | 0.97 |  |  |  |
| ω |  |  |  |  |  |  | 0.39 | **0.62** | 0.72 |  |  |  |
| NLL | 78.29 | **96.19** | 117.23 | 77.22 | **94.69** | 118.74 | 76.14 | **96.13** | 116.81 | 116.65 | **124.48** | 132.06 |
| BIC | 85.87 | **103.69** | 124.77 | 79.59 | **97.20** | 121.31 | 85.92 | **106.13** | 126.86 | 116.65 | **124.48** | 132.06 |
|  |  |  |  |  |  |  |  |  |  |  |  |  |
| ρ^2^ | 0.17 | **0.29** | 0.35 | 0.11 | **0.31** | 0.38 | 0.16 | 0.32 | 0.40 |  |  |  |

Quartiles (median in bold) of best fitting parameters for the three algorithms used to produced regressors for the imaging analysis, along with the negative log likelihood (NLL), BIC estimated evidence, and pseudo-*r^2^* measure of individual fit.

# Table S3. Activation at decision points for model-free valuation across three phases of the wayfinding task


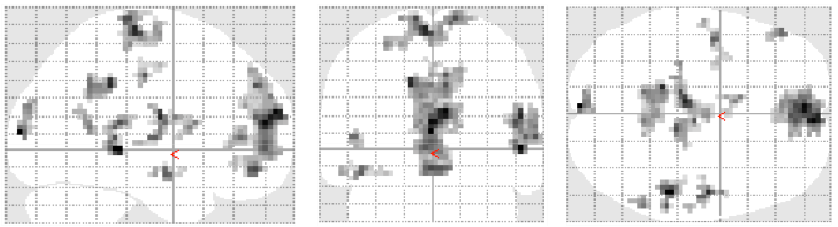


**Regions Peak Coordinates Hemi *T*_PEAK_ Z score k**

|  | **x** | **y** | **z** |  |  |  |  |
| --- | --- | --- | --- | --- | --- | --- | --- |
| Medial PFC  Superior medial gyrus | 6 | 59 | 20 | R | 6.29 | 4.86 | 524 |
| Middle orbital gyrus | -3 | 47 | -13 | L | 5.43 | 4.83 | 524 |
| Anterior Cingulate Cortex | -3 | 38 | 5 | L | 4.58 | 4.40 | 524 |
| Precentral gyrus | -6 | -25 | 68 | L | 5.92 | 4.67 | 120 |
|  | -30 | -28 | 59 | L | 4.59 | 3.89 | 120 |
|  | 12 | -31 | 74 | R | 5.33 | 4.34 | 49 |
| Caudate Nucleus | -9 | 11 | 14 | L | 4.69 | 4.11 | 29 |
| Middle temporal gyrus | 51 | -34 | -1 | R | 6.47 | 4.95 | 73 |
|  | -45 | -7 | -16 | L | 5.10 | 4.21 | 35 |
| Superior temporal gyrus | 51 | -10 | 17 | R | 5.86 | 4.64 | 52 |
|  | 60 | -22 | 2 | R | 4.09 | 4.54 | 52 |
| Retrosplenial cortex (RSC) | -15 | -40 | 35 | L | 5.85 | 4.63 | 130 |
|  | 6 | -52 | 32 | R | 4.72 | 3.98 | 130 |
|  | 6 | -19 | 41 | R | 5.22 | 4.28 | 37 |
| Cuneus | 0 | -85 | 26 | L | 4.28 | 3.84 | 63 |
| Calcarine gyrus | -3 | -94 | 8 | L | 6.49 | 4.94 | 63 |

Regions showing correlated BOLD activity with model-free values as parametric regressors across three different phases of the wayfinding task. Statistical significance was determined at the group level using a random-effect analysis. Regions listed exhibited significant peak voxels at probability threshold of *P* < 0.05 based on a FWE cluster level small volume correction (*k* = 25). Peak voxel MNI coordinates x, y, z are given in millimeters. L, left; R, right.

# Table S4. Activation at decision points for model-based valuation across three phases of the wayfinding task


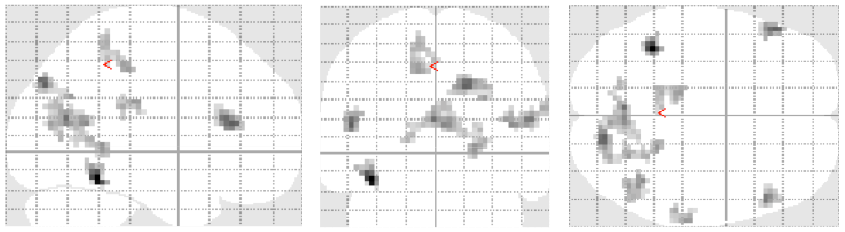


**Regions Coordinates Hemi *T*_PEAK_ Z score k**

|  | **x** | **y** | **z** |  |  |  |  |
| --- | --- | --- | --- | --- | --- | --- | --- |
| Lateral PFC  Inferior frontal gyrus | -51 | 29 | 14 | L | 5.29 | 4.32 | 97 |
| Inferior frontal gyrus | 54 | 23 | 17 | R | 4.93 | 4.11 | 35 |
| Cuneus | 15 | -79 | 35 | R | 5.26 | 4.30 | 61 |
| Precuneus | 27 | -64 | 29 | R | 4.39 | 3.77 | 61 |
| Supramarginal gyrus | 66 | -28 | 26 | R | 4.51 | 3.84 | 30 |
| Middle temporal gyrus | 42 | -55 | 14 | R | 4.69 | 3.95 | 43 |
| Fusiform gyrus | -39 | -49 | -16 | L | 6.85 | 5.13 | 36 |
| PPA  Parahippocampal gyrus | 21 | -46 | 2 | R | 4.54 | 3.85 | 153 |
| Calcarine gyrus | -3 | -67 | 17 | L | 5.08 | 4.20 | 153 |
|  | 5 | -75 | 14 | R | 4.38 | 3.75 | 153 |
| Retrospinal cortex (RSC) | -12 | -31 | 44 | L | 4.39 | 3.75 | 56 |

Regions showing correlated BOLD activity with model-based values as parametric regressors across three phases of the wayfinding task. Statistical significance was determined at the group level using a random-effect analysis. Regions listed exhibited significant peak voxels at probability threshold of *P* < 0.05 based on a FWE cluster level small volume correction (*k* = 25). Peak voxel MNI coordinates x, y, z are given in millimeters. L, left; R, right.

**References**

1 Harris, M. A., Wiener, J. M. & Wolbers, T. Aging specifically impairs switching to an allocentric navigational strategy. *Front Aging Neurosci* **4**, 29, doi:10.3389/fnagi.2012.00029 (2012).

2 Latini-Corazzini, L. *et al.* Route and survey processing of topographical memory during navigation. *Psychol Res* **74**, 545-559, doi:10.1007/s00426-010-0276-5 (2010).

3 Shikauchi, Y. & Ishii, S. Decoding the view expectation during learned maze navigation from human fronto-parietal network. *Scientific Reports* **5**, 17648, doi:10.1038/srep17648 (2015).

4 Viard, A., Doeller, C. F., Hartley, T., Bird, C. M. & Burgess, N. Anterior hippocampus and goal-directed spatial decision making. *J Neurosci* **31**, 4613-4621, doi:10.1523/jneurosci.4640-10.2011 (2011).

5 Voermans, N. C. *et al.* Interaction between the human hippocampus and the caudate nucleus during route recognition. *Neuron* **43**, 427-435, doi:10.1016/j.neuron.2004.07.009 (2004).

6 Wiener, J. M., de Condappa, O., Harris, M. A. & Wolbers, T. Maladaptive bias for extrahippocampal navigation strategies in aging humans. *J Neurosci* **33**, 6012-6017, doi:10.1523/jneurosci.0717-12.2013 (2013).

7 Vandenberg, S. G. & Kuse, A. R. Mental rotations, a group test of three-dimensional spatial visualization. *Percept Mot Skills* **47**, 599-604, doi:10.2466/pms.1978.47.2.599 (1978).

8 Caissie, A. F., Vigneau, F. & Bors, D. A. What does the Mental Rotation Test Measure? An Analysis of Item Difficulty and Item Characteristics. *The Open Psychology Journal* **2**, 8 (2009).

9 Wunderlich, K., Smittenaar, P. & Dolan, R. J. Dopamine enhances model-based over model-free choice behavior. *Neuron* **75**, 418-424, doi:10.1016/j.neuron.2012.03.042 (2012).
